# The Impact of Feed Supplementations on Asian Buffaloes: A Review

**DOI:** 10.3390/ani11072033

**Published:** 2021-07-07

**Authors:** Amirul Faiz Mohd Azmi, Hafandi Ahmad, Norhariani Mohd Nor, Yong-Meng Goh, Mohd Zamri-Saad, Md Zuki Abu Bakar, Annas Salleh, Punimin Abdullah, Anuraga Jayanegara, Hasliza Abu Hassim

**Affiliations:** 1 Department of Veterinary Preclinical Sciences, Faculty of Veterinary Medicine, Universiti Putra Malaysia, UPM Serdang, Serdang 43400, Malaysia; amirulfaizazmi@gmail.com (A.F.M.A.); hafandi@upm.edu.my (H.A.); norhariani@upm.edu.my (N.M.N.); ymgoh@upm.edu.my (Y.-M.G.); zuki@upm.edu.my (M.Z.A.B.); 2 Department of Veterinary Laboratory Diagnosis, Faculty of Veterinary Medicine, Universiti Putra Malaysia, UPM Serdang, Serdang 43400, Malaysia; mzamri@upm.edu.my (M.Z.-S.); annas@upm.edu.my (A.S.); 3 Faculty of Science and Natural Resources, Universiti Malaysia Sabah, Jalan UMS, Kota Kinabalu 88400, Malaysia; puniminabdullah@ums.edu.my; 4 Department of Nutrition and Feed Technology, Faculty of Animal Science, IPB University, Bogor 16680, Indonesia; anuraga.jayanegara@gmail.com; 5 Animal Feed and Nutrition Modelling (AFENUE) Research Group, Department of Nutrition and Feed Technology, Faculty of Animal Science, IPB University, Bogor 16680, Indonesia; 6 Laboratory of Sustainable Animal Production and Biodiversity, Institute of Tropical Agriculture and Food Security, Universiti Putra Malaysia, UPM Serdang, Serdang 43400, Malaysia

**Keywords:** bypass fat, buffalo, concentrate, performance, supplementation

## Abstract

**Simple Summary:**

Apart from feeding with forages, dietary supplementation with concentrate and rumen bypass fat is one of the feeding strategies to enhance nutrient availability and improve buffalo performance and productivity. This review paper thoroughly discussed the utilization of concentrate and bypass fat as dietary supplementation in buffalo feeding, and discussed the effects on performance, fermentation characteristics and general health of buffaloes to give better insight about the potential and challenges of dietary supplementation in buffalo diet. Based on the literature studies, it can be summarized that supplementation of concentrate and bypass fat in buffaloes may overcome the nutritional problems and improve the growth performance, health status, rumen environment and carcass traits.

**Abstract:**

With the increase in the global buffalo herd, the use of supplementation in the ruminant feeding has become an important area for many researchers who are looking for an isocaloric and isonitrogenous diet to improve production parameters. In order to improve the performance of the Asian water buffalo, the optimal balance of all nutrients, including energy and protein, are important as macronutrients. Dietary supplementation is one of the alternatives to enhance the essential nutrient content in the buffalo diet and to improve the rumen metabolism of the animal. Researchers have found that supplementation of concentrate and rumen bypass fat could change growth performance and carcass traits without causing any adverse effects on the buffalo growth. Some studies showed that dry matter intake, body condition score and some blood parameters and hormones related to growth responded positively to concentrate and rumen bypass fat supplementation. In addition, changes of feeding management by adding the supplement to the ruminant basal diet helped to increase the profit of the local farmers due to the increased performance and productivity of the animals. Nevertheless, the effects of dietary supplementation on the performance of ruminants are inconsistent. Thus, its long-term effects on the health and productivity of buffaloes still need to be further investigated.

## 1. Introduction

The Asian water buffalo (*Bubalus bubalis*) is an important animal resource in a minimum of 67 developing countries. Many people rely on this species for their livelihoods [1]. It provides economic value from its meat, milk and leather, and especially draft power [2]. An estimation by Scherf [3] revealed that more than 2 billion people depend on approximately 200 million heads of buffaloes, which is more than any other domesticated animal. The majority of buffaloes operate in close association with humans on small farms, for whom these animals are also their largest capital asset. Based on morphological and behavioral criteria, there are two subspecies of domesticated water buffaloes, the swamp (*Bubalus bubalis carabanesis*) and the river or Murrah (*Bubalus bubalis bubalis*) buffaloes [4,5]. Crossbreds between Murrah and Swamp (*Bubalus sp.*) buffaloes are found in some parts of Asia, especially in China, Indonesia, the Philippines, Thailand, Vietnam and Malaysia [6].

Currently, buffalo management practices are intrinsically correlated to the status of buffalo health, production and welfare. In order to ensure the optimum health of the animals and the organized production of high-quality and safe animal products (meat, milk and dairy products), a proper diet ration arguably represents the easiest strategy that can be implemented by farmers at the farm level. Both the meat and the dairy buffalos’ industries have made significant advances in animal management, husbandry, genetics and nutrition. However, the current climate change phenomenon has caused a reduction of the availability of rangeland pastures and forages, especially for animals reared under the free-grazing system [7]. According to Henry et al. [8], extreme and fluctuating temperature may impair the optimum health and quality of life of animals. Furthermore, changes of the economic patterns around the world due to COVID-19 issues and the increasing demand for livestock as well as animal products from the developing countries trigger all the stakeholders involved in ruminant production to reconsider the strategic use of nutrition for enhancing animal health and production. Indeed, the livestock industry must find alternative nutritional strategies which meet the demand of consumers for economical animal products that are produced in clean, halal, green and ethical manners [9]. From our literature review, we found an abundance of scientific trial studies on animals (in vitro and in vivo) that indicated reliable and cost-effective approaches for increasing ruminant profitability through optimizing the composition of the feed nutrients and with the addition of supplementation [7].

Nowadays, the buffalo industry in Malaysia is starting to gain some attention as there are currently more studies on buffalo production, especially on how to improve it. This is in line with what has been carried out elsewhere, especially in other Southeast Asian countries. We therefore propose an approach using a suitable diet of a combination of basal diet with supplementation aimed for growing buffaloes. Hence, in this review article, we discussed the challenges of the buffalo industry, the requirements for energy and protein for buffalo growth, the potential of supplementation in the buffalo diet and the effects of the supplemented diet on rumen fermentation characteristics, growth performance, quality of the buffalo meat and the economics of the feed ration in ruminants. Our findings would allow specific dietary supplementation-based strategies to be established, which could efficiently enhance the health, welfare and longevity of the buffalo.

## 2. Characteristics of Asian Water Buffalo

### 2.1. Morphology and Genetics

In the field, river and swamp buffaloes can be differentiated based on their morphology and behavior. Swamp buffaloes are ash or dark grey with a white chevron line on the neck, either one or two stripes, and have socks, while the tip of the tails and the horns are swept backwards [10,11]. They prefer to wallow in the marshland and mud, and have large feet with slow steady movement that make them well-suited for paddy land preparation in swampy waterlogged rice fields [12]. On the other hand, river (Murrah) buffaloes have black bodies with tightly and forwardly curled horns. They prefer to wallow in clean water [10]. The crossbred buffaloes have the same morphology as the Murrah but are smaller than the Murrah and bigger than the Swamp buffaloes.

The chromosome number also differs between Swamp and Murrah, with river 2n = 50 and swamp 2n = 48 [11], owing to the fusion of chromosomes 4p and 9 [13]. The river and swamp buffaloes are also genetically distinct, as confirmed by the variations in allozymes, sex-linked, autosomal DNA markers, mitochondrial DNA sequences, microsatellite variation and a comparison with protein-coding loci [11,14,15]. Malaysian swamp buffaloes from the peninsula and Borneo region such as Sabah and Sarawak states were studied in this work. The peninsular and the Borneo swamp buffaloes were found to be genetically distinct, hence paving the way for the possibility of crosses between them to improve the Malaysian swamp buffaloes [6]. In addition, a prior study in Malaysia had reported that phylogenetic tree and mtDNA analysis on Swamp buffaloes were genetically conserved and the crossbreds were dominantly Swamp according to the maternal lineage using d-loop mtDNA [6].

On the other hand, crossbred buffaloes are the result of a combination of the two genetic types, but showing 80% dominant characteristics of the Murrah breed [6]. Crosses between river and swamp buffalo are fertile, despite having 2n = 49 chromosomes in *F*_1_ and *F*_2_ offspring. The crossbred buffaloes show better growth, larger body size and much improved draft power compared to the local swamp buffaloes [16]. Furthermore, the crossbred buffaloes are capable of yielding extra milk production, with an average of between 4.0 and 1.94 kg/day [17]. In fact, crossbred buffaloes are much more improved in terms of birthweight, age at maturity and first calving, duration of heat and period of inter-calving [18]. Indeed, with improved feeding, the crosses were recorded to grow 40% faster, with significantly improved meat quality than the Swamp buffaloes [19].

### 2.2. Distribution and Use

**Figure 1:**
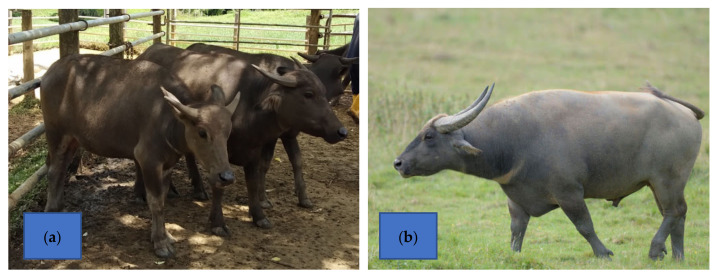
The swamp buffaloes. (**a**) Calves Swamp buffalo, (**b**) adult Swamp buffalo. Reprinted with permission from ref. [21]. Copyright 2021 Mohd Azmi et al.

The indigenous breed of buffalo in East and Southeast Asia is the Swamp buffalo (*Bubalus bubalis carabanesis*). It is largely concentrated in Southeast Asia and Southern China (Figure 1). In China, the Swamp buffaloes have adapted to a range of climates, altitudes and temperatures [20]. Therefore, the Swamp buffaloes could be found in both low- and high-lands. Traditionally, the Swamp buffalo is used mainly for draught power, particularly for ploughing paddy fields and transportation, but it is also used to supply meat for human consumption. Until now, some oil palm estates are still using Swamp buffaloes as draught animal to pull carts carrying oil palm bunches. Occasionally, Swamp buffaloes’ milk is used to make dairy products such as yoghurt and mozzarella cheese.

**Figure 2:**
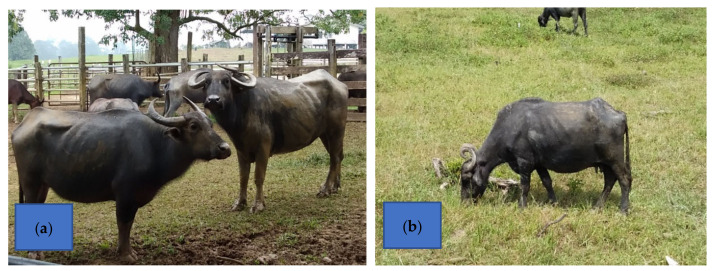
The crossbred and pure-bred Murrah buffaloes. (**a**) Crossbred buffalo and (**b**) pure-bred Murrah buffalo. Reprinted with permission from ref. [21]. Copyright 2021 Mohd Azmi et al.

River or Murrah buffaloes (*Bubalus bubalis bubalis*) are mainly found in South Asia, with the highest distributions in Pakistan, India, the Middle East and Italy (Figure 2). They are primarily reared for their milk typified by the high contents of fat and dry matter. Furthermore, buffalo milk has lower cholesterol content, but compared to cow’s milk, it has more calories and fat. Thus, it is used to produce high-value thick and creamy cheese, yoghurt and ghee [20]. Murrah buffaloes are also used to improve buffalo milk production in many other countries such as Egypt and Bulgaria [22]. According to Hamid et al. [23], the Murrah buffaloes have been described as the “Asian tractors” and serve the purpose of meat, milk and work. In contrast to the Swamp buffaloes, river buffaloes are more aggressive, with temperamental instability [24].

The multipurpose crossbred buffaloes are mainly found in several parts of Asia, especially in China, Indonesia, the Philippines, Thailand and Vietnam. They are used to provide draught power and meat in rice-growing areas and milk in other regions [25]. Now, crossbreeding of buffaloes is practiced in almost all countries where Swamp buffaloes are found, such as China, Burma, Thailand, the Philippines, Malaysia and Sri Lanka, with the aim of improving the milk yield and the animal size for work in the field [26].

## 3. Challenges in Buffalo Production Systems

An alarming decline in the buffalo population of Southeast Asia has happened over the past two decades at an average rate of 1.2 percent per year. This is due to several factors: (i) poor market demand for buffalo products [27], (ii) high rate of slaughter coupled with insufficient input for research and development [28], (iii) increased agricultural mechanization that made the Swamp buffalo redundant, (iv) a myth against the quality of buffalo meat [29], (v) poor reproductive performance coupled with poor responses to the biotechnology currently available, such as embryo transfer technology and artificial insemination, which prevented sufficient proliferation of the buffalo [17], and above all, (vi) a lack of knowledge on farm and feed management, resulting in a rapid decline in the number of buffaloes.

Furthermore, a few other factors have been cited as possible constrains that had contributed to the low output from beef and mutton producers. These factors include the inadequacy of land suitable for grazing to sustain significant livestock breeding populations, low supply of quality breeding stocks, erratic supply of high nutritional value feed and lack of an effective system of marketing. Taking cues from the poultry industry, the beef industry must promote ready supplies for the breeding and fattening of the production stock, ensuring the continuous supply of reasonably priced buffalo feed and creating an effective marketing network [30].

In Asia, in accordance with the environment, soil and socioeconomic opportunities, buffalo production systems vary widely [31]. The semi-intensive production system practiced by smallholder farmers is currently focused upon by many developing countries for the ruminant industry, mainly for cattle and buffalo. According to Saadullah [31], buffaloes in Asia that are mostly under the semi-intensive system are kept mainly for specific purposes, such as for meat and milk production. Approximately, 11%, 5% and 84% of the smallholder farmers reared buffalo for milk, meat and for both milk and meat, respectively [32]. Recently, the extensive production system is used through integrating ruminants, including buffaloes with oil palm or rubber plantations [30], where large-scale pasture lands and green forages or grasses are available [33] that allow the animals to graze for an average of 6 to 8 h daily [34]. However, most buffalo farms practice the semi-extensive grazing system in oil palm or paddy field areas without supplementation [35].

South and Southeast Asia have many marshlands and rivers which are suitable for raising buffaloes. Moreover, improvements in feeding management have influenced the growth performances of buffaloes in countries such as India, Brazil, the Philippines and Malaysia [35,36]. In general, indigenous Swamp and crossbred buffaloes in Southeast Asia are low in numbers, and this has affected the production of dairy and meat. Furthermore, longer puberty age, seasonality of breeding, longer calving interval, high calf mortality and poor genetic pool, nutrition and management practices [37,38,39] have all influenced the productivity.

## 4. Feeding and Nutritional Management for Buffalo Production

### 4.1. Nutrient Requirements and Utilization of Buffalo

Like other ruminants, buffaloes obtain their energy and protein in the form of volatile fatty acids and microbial proteins from fermentation end products. Based on research studies on the nutritional requirements and digestive physiology of buffaloes, it was concluded that buffaloes underwent relatively higher ruminal degradation of both protein and fiber [40] as compared to cattle [41]. This unique ability, particularly the better fermentation of fibers in buffaloes in temperate countries, is the consequence of adaptation following years of being fed with low-quality and highly fibrous diets [42]. According to Manish [43], the protein and energy demands of buffaloes in Asia were being met by feeding roughages containing high levels of lignocellulosic materials, namely cellulose, hemicellulose, lignin and low levels of fermentable carbohydrates and proteins. Indeed, the irregular and inadequate availability of quality feedstuffs and their utilization had been reported as the main causes of the poor performance of buffaloes in Asia [44]. In contrast, developed countries that produce a large number of meat and dairy animals place much emphasis on improving energy and protein levels of animal feed as well as on developing a specific model of nutrient requirements in buffaloes. These were reported by various studies, where the levels of energy and protein requirements varied in buffalo diets during lactation and growth [41,42,45]. Nevertheless, limited strategic studies were performed to establish the protein and energy needs of buffaloes in Asia, particularly on assessing their effects on different physiological stages and on the performances of buffaloes of various breeds.

In general, buffaloes need optimal nutrient requirements such as protein, fat, vitamins, minerals and water in order to maintain life, to reproduce and to enhance productivity. These requirements are influenced by many factors [46,47]. They include:

1. Animal-based factors, such as the physiological status (growth), age and body weight of animal, production line, traits of digestive system and health status [48,49].
2. Ration-associated factors, including the feeds that are used in the ration and the nutritional, physical and chemical composition status of the feed [50].
3. Environmental-related factors, particularly the climate, air temperature and feeding system [51].

Currently, there is paucity of information regarding the energy requirements of growing, breeding, lactating or working buffaloes [52]. Therefore, the nutrient requirements for cattle have always been adopted for buffalo feeding following the recommendations by NRC [53] and AFRC [54].

Energy is typically derived from carbohydrates, namely starch, cellulose and fat [47]. The nature of the buffalo digestive system physiology makes the cellulose in roughages, a rather cheap energy source, important [55]. The recommended values of energy requirements for buffaloes differ depending on their stages and physiological conditions. Estimations of the energy requirements of buffalo gain in the literature varied from 0.78 to 2.23 TDN/g gain [52], and for the lactating stage it was 1.97 TDN/g gain [48]. In addition, the amounts of feedstuffs offered to buffaloes to meet their requirements were also linked to various characteristics, such as the type, quantity, quality and presentation of the feed [52]. Therefore, besides feeding with low-quality roughage, the high demand for energy by growing buffaloes should be fulfilled by supplementing with a mixture of quality roughage and grains that contained an abundance of energy [56]. Furthermore, adequate supplementation of fat was capable of increasing the concentration of energy in the animals, which could also enhance the percentage of fat in the milk as well as the quality of the meat [57].

Protein is an important substance for growth and development of muscles, nerves and other tissues. It is also important for the repair of aged tissues, fetus development and the production of meat and milk [58]. Ammonia is required for the growth of rumen microorganisms before optimal microbial protein synthesis is achieved by supplementation with adequate levels of protein and non-protein compounds [59]. Other studies reported earlier that there was a large variation in the values for protein requirements in buffaloes [48,52]. The estimated range of digestible crude proteins needed by buffalo for maintenance and growth was 1.11 to 5.05 g/kg metabolic body size ^0.75^ and 0.30 to 0.45 g/g gain, respectively [52]. Following a low level of protein or energy supply to buffaloes, the demand for proteins was met by a low supply of medium-quality pastures and fodder. Therefore, growing, pregnant and lactating buffaloes should be fed with meadow grass and leguminous forage supplemented with concentrate, grain or oil seed cakes [46]. This could prevent protein insufficiency that might lead to declines in appetite and feed consumption of the animals, a negative utilization of feed and a reduction in cellulose digestion [55].

### 4.2. Roughage Feeding

It is clear that buffaloes’ main diet consists of roughages such as grass, legumes and straw. Most of the buffaloes are fed on tropical grasses as the primary source of nutrients. The roughages are fed either fresh as the grazing pasture or dried such as in a cut and carry system or conserved as hay or silage. Thus, a relatively low cost of digestible energy from grasses is provided to the buffaloes, mainly in the form of fibrous compounds [60]. Nevertheless, the roughage that forms the basis of a feed ration should be of good quality, have both nutritional and hygienic qualities and be capable of meeting at least the total maintenance requirements. Unfortunately, tropical grasses for grazing buffaloes infrequently represent a balanced diet, since they have constraints on one or more nutrients that may limit the intake of forage, digestibility or the metabolism of the absorbed substrates [61]. Therefore, an addition of concentrate in the diet should be practiced in buffaloes’ feeding to ensure a balanced ration, and nutrients are provided to meet the buffaloes’ requirements [62].

To improve buffalo performance, the utilization of pastures in any season as the main nutrients is not considered to be optimal [61]. Thus, selecting mature, dried foliage and stems of grasses ensures supply of a low protein level of less than three percent of crude protein. Furthermore, the grasses have been variably leached of soluble components, including minerals, proteins, sugar and starchy carbohydrates that are needed for efficient fermentative digestion in ruminants. On the other hand, too much intake of non-fibrous feed can change the environment of the rumen, and in the long term, leads to serious feed digestion problems, including reducing feed intake, which leads to ketosis, weight loss and a reduction in milk yield. According to Figueiras [61], unbalanced energy to protein ratios were recorded in tropical grasses, with relative energy surplus. Thus, the use of this poor-quality forage without a supplementary diet contributed to the low production of the ruminant meat industry in developing countries. In fact, a study in Brazil and Australia revealed that using a proper feed supplementation program improved the body weights of ruminants [60]. For this reason, there is a need to recognize tropical pastures as having limitations of nutrients and to solve this or to change to feeding regimes that may lead to improved performances of the animals and the overall production efficiency [63].

### 4.3. Supplementation Strategy

Low growth performance, poor reproductive performance and milk yield have been reported in buffaloes in other studies [64,65]. Indeed, the poor performance reported by those studies correlated with poor dry matter intake and weight gain and longer calving intervals [64,65]. In many parts of Asia, inadequate and irregular availability of quality feedstuffs and their utilization are the main causes of the poor performance of buffaloes [44]. A report stated that buffaloes in Southeast Asia were mainly fed with hay or forages high in lignin and low in fermentable protein and carbohydrate contents [44]. Many strategies have been trialed in order to improve the nutrient supply and utilization in buffaloes, with varying degrees of success. These are: (1) utilization of available feed resources such as local plants that have high crude protein or energy content, (2) use of industrial and agricultural by products, (3) dietary addition of concentrate, fermentation modifiers and vitamins and (4) usage of ruminally protected dietary fat and protein sources, which have shown significant potentials to improve growth, reproduction and milk yield of buffaloes [44]. However, in order to choose the best strategy to improve buffalo performance, the farmer should identify the main problem causing the low growth performance of the animal.

In the buffaloes’ diet, the roughage should often be complemented with concentrate, grains or agro-industrial products as supplements. The supplements should be fed only to fulfil the additional requirements for improving growth, pregnancy and milk production. Therefore, feed supplementation programs should concentrate mainly on the establishment of a diet that contains balanced nutrients by increasing the energy content in the diet as well as by increasing the dry matter intake through the addition of supplements. Indeed, a supplemented diet in the tropics should focus primarily on protein and fat supplementations in order to provide optimum energy for better growth performance of the animals [63,66]. This would allow for utilization of the relative excess of energy from the supplemented diet to be converted into body weight gain [67]. Balancing the fat and protein nutrition through supplementation is one of the strategies for increasing production in ruminants with high-energy diet requirements, such as young post-weaning animals, animals in the last trimester of pregnancy and lactating animals.

In most regions of developing countries, it is not practical to identify the deficient micronutrients and macronutrients in pastures or other forages, as these vary from site to site and year to year. Furthermore, they are also influenced by the pattern of fertilizer application and the weather conditions. The alternative is through providing buffaloes with a supplemented diet, and the supplementation involves providing energy and protein to satisfy the requirements for efficient digestion in the rumen. Diet with a proper supplementation is able to provide optimal ammonia nitrogen and good fatty acid in the rumen. Palatable and tasty feed with a well-balanced ratio of protein and fat as additional energy sources is the best way to increase milk production and live weight, maintaining health and enhancing fertility. This has influenced the changes in supplementation utilization, since a lack of knowledge on feeding management would affect animal production and nutritional performance [63,66]. However, comparative analyses regarding the combination of energy and protein supplementation on buffalo performance in the tropics remain scarce. Therefore, extensive studies are needed to assess the impacts of supplements on grazing buffaloes in the tropics, particularly on the intake, ruminal fermentation pattern and the quality of the meat.

### 4.4. Types of Supplementation in Buffalo Diets

Supplemented feed offers a promising way to enhance the overall health and performance of buffaloes. In contrast to a nutritionally complete ration, supplemental diets are intended to provide an additional source of energy and protein when forage quantity and quality are inadequate to meet the desired performance [68]. Nutritional husbandry of domestic buffalo often contains high energy and protein supplements in combination with roughage to increase the growth rate of sub-adult animals [69] and to enhance the digestibility of forage diets [70]. Supplementation strategies are essential in designing the feeding programs for this species. In fact, supplementation with larger amounts of energy-rich feeds with a source of protein and fat could reduce the time taken to prepare buffaloes for the market, thus increasing profitability, such as those reported for cattle that consumed low-digestibility forages with energy and protein supplements [71].

Concentrate is one of the dietary supplements that supply protein to animals. Many experiments have demonstrated the benefits of supplementing dietary protein meals or concentrate to ruminants that are fed poor-quality forage [72,73,74]. In fact, in developing countries, low cattle and buffalo productions are primarily due to limited supplies of nutrients in high forage-based rations [75]. Therefore, it is important to identify new feed sources and technologies for cattle and buffalo production systems. Recently, there are several varieties of concentrate ingredients that have been used by smallholder farmers, namely maize meal, cassava powder, rice bran and mixtures of these feedstuffs in ruminant production [75], such as cattle and buffalo. However, due to inadequate information on the nutritional value, digestibility and characteristics of the rumen fermentation, the benefits of using these feeds for buffalo are not well-understood. This is especially true since previous studies had reported that different proportions of concentrate feeds differed substantially in their rumen fermentation characteristics [76]. Nevertheless, a high starch content in the concentrate is important in ruminant nutrition because it is cost-effective, contains protein sources and has been proven able to influence rumen function and digestion of nutrients [77,78].

The technology of bypass nutrients such as rumen bypass fat has been implemented in feed management through passive rumen manipulation [79]. It is also known as rumen-protected fat, inert fat or rumen bypass fat [80]. Bypass fat is the supplement that escapes rumen degradation as it is being protected from microbial fermentation, biohydrogenation and remains insoluble at normal rumen pH. However, it is easily digested and absorbed in the lower gastrointestinal tract at the abomasum and small intestine, respectively [81]. Therefore, it might be beneficial for ruminants to be fed with rumen bypass fat as a supplementary diet that is absorbed readily from the lower digestive tract. There are two types of rumen bypass fat supplements commercially used by farmers, namely natural bypass fat (e.g., cotton, roasted soybeans, sunflower and canola whole oil seeds) and chemically prepared bypass fat (e.g., crystalline or prilled fatty acids, formaldehyde-treated protein-encapsulated fatty acid, fatty acyl amide and calcium salts of long-chain fatty acids) [82]. The calcium salt-coated method on fatty acid of vegetables has been reported to be a more accessible bypass fat to all types of farmers. It has also been proven to be a cost-effective technology compared to other rumen bypass fats [83]. A few studies reported improvements on the growth and nutrient utilization of buffalo calves after being fed with bypass nutrients [84,85]. Integration of fat supplement into the diet enhances the growth potential of buffalo calves [79]. Studies have reported that high fat supplementation, such as RPF, could also enhance fiber digestibility of various fibrous feedstuff due to the high hydrolysis rate in rumen (85% to 95%) [81,82]. This supplement can also improve energy efficiency, as a result of reduced production of methane from the rumen and direct use of long-chain fatty acids [86]. Many studies recommended that the ration of high producing and growing animals should contain between 3% to 6% fat in the total ration DM, in order to obtain the beneficial effects [82]. Meanwhile, feeding more than 9% rumen bypass fat would not be beneficial for the growth (feed intake) and lactation (milk yield) of the animals [82].

## 5. Effect of Dietary Supplementation on Buffalo Production

**Figure 3:**
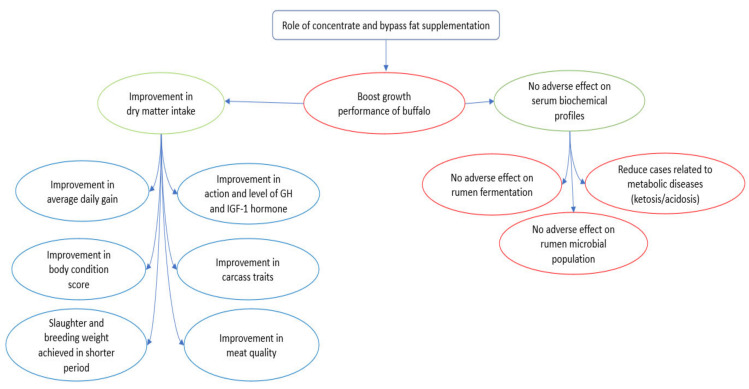
An overview of the role of concentrate and bypass fat supplementation in buffalo nutrition.

The advantages of high energy and protein in dietary supplements can be the explanation for the improvements in the growth and fattening of ruminants. Indeed, it has been shown that apart from improvement of the growth performance upon supplementation of concentrate and bypass fat, the improvement in dry matter intake may further enhance the average daily gain, growth hormones (e.g., GH and IGF-I), body condition score, carcass traits, meat quality and breeding performance of buffaloes [21,79,87]. In addition, the supplementation of both concentrates and bypass fat did not cause any adverse effects on serum biochemical profiles, rumen fermentation and microbial population [21,28,33,34,35]. An overview of the role of these concentrate and bypass fat supplementations in buffalo nutrition is presented in Figure 3.

### 5.1. Growth Performance

**Figure 4:**
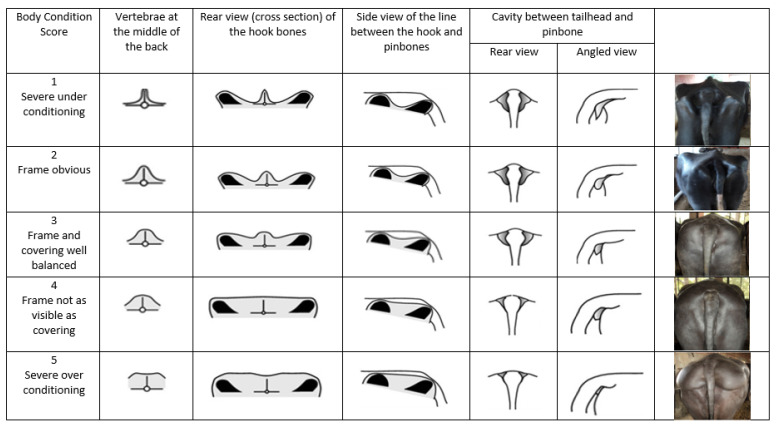
Body condition scoring chart for buffalo in a scale using 1.0 increments. Reprinted with permission from ref. [87]. Copyright 2017 Singh et al.

The body weight gain and the body condition score (BCS) of buffalo calves after weaning represent the growth vigor of the animal. This is a substantial feature in the selection of animals [88]. The body condition score of a buffalo is classified as a subjective scoring method to assess the outer appearance of the animal, which interacts with its body fat to provide a better understanding of the biological relationships between body fat, reproductive performance and production of milk. These could assist in the implementation of optimal management practices to achieve maximum production and to preserve better health status [87]. Indeed, the BCS may also provide an immediate evaluation of the body condition of the animal and can be readily integrated into operational decision-making [89]. The use of BCS started in studies of ewes, beef cattle and Holstein dairy cows in 1961, 1976 and 1989, respectively. It used a 0 to 5 scale with a chart developed for references of body condition scoring [87]. In Pakistan, there was a study on Nilli Ravi buffalo that assessed the BCS by using a linear scale of 1 to 9 (1–3 for thin, 4–6 for average and 7–9 for fat), where the scoring was performed visually by assessing the covering of fat over the tail head, rump, sacral bone and loin and withers area [90]. However, the BCS of buffaloes was improved and established in India [87] using a 1 to 5 scale for assessing the animals (Figure 4). India has the highest buffalo population in the world and shows dramatic increases of population numbers yearly [91]. In addition, it is the native tract for the best buffalo breeds of the world [92]. The improved chart for BCS of buffalo with a 1 to 5 scale using 0.25 increments has been widely used in Asian countries [92]. The BCS score assessment was carried out by taking into consideration the anatomical features and amounts of fat reserves at various skeletal checkpoints. Validation of the precision of the BCS score had been carried out via ultrasonic measurements of subcutaneous fat [92]. Thus, the use of the BCS 1 to 5 score is suitable for assessing the reproduction and production status of buffaloes. Furthermore, the most widely used criterion is also to determine the growth of animals by assessing their body weights. Body weight is significantly associated with the types of feed and ration offered.

According to Vahora et al. [79], improvements in the average daily weight gain, body length, height and heart girt in buffaloes fed with a basal diet were significantly associated with the incorporation of protein and fat supplements. Furthermore, offering supplementation at 0.6 kg/animal/day to grazing buffaloes after weaning for a period of two years enabled the calves to reach an average of 578.38 kg body weight, with improved body condition scores [36]. Similarly, supplementing beef heifers with dietary energy supplements increased the average daily gain and utilization of energy from native forage which contained low-quality nutrients [93]. Sawyer et al. [94] also showed that supplementing with a low ratio of energy at 40 g/day of crude protein might potentially replace greater quantities (160 g/day of crude protein) while still maintaining rumen function. However, animals grazing on low-quality dormant range and fed with a supplementation had no change in body weight during pregnancy, breeding and longevity compared to those feds grazing without supplement or a lower rumen undegradable plant-based protein supplement [95]. However, adding supplemental fat in the ration at a rate of 5–7% dry matter (DM) resulted in an improved lamb weight at 15% to 20% [96]. Ngidi et al. [97] reported that the apparent digestibility of fat increased, whereas true digestibility lessened when fat was added at up to 8% of diet DM. Meanwhile, Kumar and Thakur [98] concluded that supplementation of bypass fat at 2.5% to 4% of dry matter intake increased average daily gain and feed conversion ratio in buffalo calves and improved the growth performance without an adverse effect on nutrient utilization. It was concluded that addition of 2% to 4% of fat potentially stimulated feed intake and increased ruminant’s digestibility energy intake [99].

### 5.2. Serum Biochemistry and Hormone Profiles

The main function of blood in the body is to maintain the physiological equilibrium, but many physiological conditions may alter this equilibrium [100]. Blood contains a myriad of constituents that provide a valuable medium for clinical investigations and nutritional evaluations of an organism [101]. Serum biochemicals can be affected by age, nutrition, physiological status, sex, genetics, environmental factors and stresses, such as diseases and transportation [102]. The consequences of these variables can be measured by evaluating the physiological responses, since it is known that environmental and nutritional factors predispose animals to the occurrence of disease and decrease animal productivity. Such deviations from normal alter animal body constituents, especially the body fluids, thus health risk conditions can be well-understood by evaluating blood components. Disease incidence and malnutrition could be diagnosed by analyzing the deviations of the various hematological and serum biochemical parameters from the normal reference values [103]. In fact, these values are used to compare and interpret the metabolic state or condition of animals as a reference point [104].

In the ruminant industry, analysis of blood metabolic profiles for assessing the nutritional and health status of goats, cows and buffaloes are not widely used [105], although the health and nutritional status of animals are important. However, the conventional and common practices used to evaluate the nutritional status of animals include the body condition scoring and the gain of body weights. Therefore, the use of blood metabolites is less common in assessing nutritional status of ruminants, especially among smallholder farmers in developing countries [106,107]. Needless to say, there are some blood metabolites that are related to the nutritional status of ruminants, and they reflect the animal’s response to nutrition. They include blood total protein, cholesterol, triglyceride, glucose and urea.

Blood metabolites could be used regularly, objectively and reliably to assess the nutritional status of animals. However, the use of blood metabolite analysis in field buffalo and cattle is rare, particularly in developing countries such as Southern Africa and India due to the high cost of analyzing the samples, lack of equipment and expertise [106,107]. Similarly, the use of blood metabolites is quite uncommon in the field of buffalo management. This is unfortunate since several factors, namely the animal physiological status, nutrition, breed, age and season, are found to affect the blood metabolites, and in combination with data from body condition scores and body weights, blood metabolite analysis increases the accuracy of assessing the nutritional and welfare states of the ruminant population [107]. The success of the blood metabolite profile test alone is limited because several non-dietary factors also influence the concentrations of blood metabolites, such as herd origin, lactation stage, milk yield and season of the year [108].

According to Campanile et al. [109], the buffalo heifers fed *Brachiaria* hay with concentrate (high-energy diet) showed similarities in non-esterified fatty acid and triglyceride levels when compared to the group fed a low-energy diet (without concentrate) (0.53 vs. 0.47 mmol/L, 17.1 vs. 18.7 mg/dL, respectively), but there were significant increments in glucose, total cholesterol and high-density lipoprotein (90.5 vs. 73.6 mg/mL, 80.4 vs. 58.7 mg/dL, 64.0 vs. 45.4 mg/dL, respectively). Bertoni et al. [110] have revealed that buffaloes fed isonitrogenous diets with different energy contents showed constant blood urea content regardless of the different diets, while cattle with similar treatment showed a significant decrease in blood urea with increasing dietary energy. This indicated the decline in ammonia content in the rumen of cattle as a result of the limited ability to recycle blood urea into the rumen. On the other hand, blood urea nitrogen of buffaloes would be at optimum range following feeding with different energy content diets that ranged between 7.00 and 8.50 g/dL [111]. Tiwari et al. [112] reported that the normal glucose levels in growing buffaloes and Holstein cattle were 51 to 64 and 74 to 76 mg/dL respectively, when provided concentrate and roughages at an equal ratio. Other studies reported that Murrah buffalo fed a basal diet with concentrate supplementation and a mixture of concentrate with bypass fat supplementation had no effect on the blood urea nitrogen (BUN) (49.30 vs. 50.41, mg/L), total protein (7.57 vs. 7.75, g/dL), albumin (2.71 vs. 2.84, g/dL), globulin (4.86 vs. 4.91, g/dL) and cholesterol (106.27 vs. 106.36, mg/dL) levels [113]. However, the buffaloes that were fed with supplement bypass fat showed slightly increased high-density lipoprotein and calcium levels as compared to animals fed without the bypass fat (65.55 vs. 57.22 mg/dL and 7.03 vs. 5.70 mg/dL, respectively) [113,114]. Nevertheless, the buffaloes that were supplemented with bypass fat showed a slight decrement of blood glucose, non-esterified fatty acid and low-density lipoprotein levels as compared to animals fed without the bypass fat (63.50 vs. 65.98 mg/dL, 0.66 vs. 0.93 mmol/L, 33.66 vs. 37.49 mg/dL, respectively) [113].

Similarly, hormonal profiles can also be used to determine the health and nutritional status of ruminants. Animal growth is influenced by many hormones, blood metabolites and growth factors acting both in an endocrine and an autocrine manner and requires the coordinated actions of several hormones, such as growth hormone (GH) and insulin-like growth factor-I (IGF-I) [115]. The somatropic axis is the important hormonal system for growth development of animals. It consists of GH, IGF-I, carrier proteins and receptors [116]. The hormones of GH and IGF-I have both independent characteristic and combined impacts on ruminant metabolism and production. The growth hormone is synthesized in the pituitary gland and acts directly on the liver and adipose tissue to regulate gluconeogenesis, protein synthesis, lipogenesis, lipolysis and insulin secretion by binding to the growth hormone receptor (GHR) [117,118,119]. On the other hand, IGF-I is a critical somatomedin that is synthesized in the liver. It plays an important role in some physiological processes, contributes to improved feed conversion rate and increases protein synthesis [120]. The IGF-I binds to insulin-like growth factor binding protein-3 (IGFBP-3) to influence the growth, development and reproduction in animals [121]. Meanwhile, the existence of the axis between GH and IGF-I has played a vital role in the regulation of metabolism. GHR combines with GH to stimulate a series of metabolic activities by producing IGF-I in the target tissues, especially in the liver [122,123].

In some cases, nutritional factors are critical regulators of IGFs [124]. Deficiency in either energy or protein intake significantly decreases the IGF-I levels [124]. Similarly, Clemmons et al. [125] reported that low energy in the diet caused the IGF-I level to decrease 4-fold. In fact, limiting the energy in the diet increases the GH levels and reduces the IGF-I secretion [126,127]. Meanwhile, over-consumption increases the IGF-I, although excess calories are not nearly as strong a stimulus as nutritional restriction [128]. A short-term feeding study on protein deprivation revealed a potent and independent role of protein on the IGF-I levels. Deficiency of essential amino acids has a severe depressing effect on the IGF-I levels [124]. However, a high-carbohydrate diet increases the IGF-I levels relative to a high-fat diet, possibly by maintaining hepatic sensitivity to GH [129]. Several researchers have recorded that yaks and buffaloes have evolved and developed complex strategies to respond to the deficiencies of nutrition and hypoxia stress [130,131,132].

According to Campanile et al. [109], buffalo heifers fed with *Brachiaria* hay and supplemented with concentrate (high-energy diet) showed increases in GH and IGF-1 levels compared to buffalo fed with a basal diet without concentrate (low-energy diet) (6.3 vs. 5.6 pg/mL, 95.5 vs. 79.1 ng/mL, respectively). Other studies also revealed that Murrah buffalo increased IGF-1 and remain constant in GH levels when fed a diet supplemented with a mixture of concentrate with bypass fat when compared to being fed with a diet supplemented solely by concentrate (119.10 vs. 116.24 ng/mL, 4.91 vs. 4.93 ng/mL, respectively) [113,133]. Even though a few studies have shown the effects of dietary fat and protein supplements on the serum biochemistry profiles as well as the hormonal profiles in the blood, unfortunately, limited work has been performed on the comparison of blood and hormonal profiles between buffalo breeds.

### 5.3. Rumen Fermentation Pattern

Rumen digestion is fundamentally a fermentation process within the rumen by various types of bacteria, protozoa and fungi [134]. These microbes are responsible for the breakdown of feed and water intake into volatile fatty acids (VFA), gases (i.e., methane, carbon dioxide) and microbial proteins that are useful for the animal. For the rumen microbes to function properly, the rumen environment parameters including pH, temperature and moisture must be maintained [134]. Therefore, the outcome of rumen fermentation depends on adequate nutrition with respect to composition and quality of feedstuffs, which is reflected in the voluntary intake and digestibility of ruminants [135].

Buffaloes are like cattle which utilize micro-organisms in the rumen to digest the feed. It has been reported that many farms until today fed their buffaloes using cow requirements as a reference point [136]. This might be due to the similarities in rumen fermentation characteristics between buffalo and cow. According to Smith et al. [137], both animals have similarities in heat tolerance, milk composition and ability to utilize highly fibrous feed [138,139]. Nevertheless, studies also revealed that buffaloes indeed have higher forage digestibility due to higher populations of cellulolytic bacteria, total fungi and lower protozoa numbers in their rumen, as compared to cattle rumen [140,141]. Therefore, it is important to understand the rumen fermentation and the ruminal microbial differences between buffaloes and cows when formulating a feeding regime for them [138].

Changes in diet, from forage to high-protein diet, can affect the fermentation process of ruminants such as cattle and buffalo. The increase in dietary crude protein (CP) leads to increased concentration of total rumen volatile fatty acid (VFA), which is consistent with the improved degradability following increased bacterial populations and microbial enzyme activities [142,143]. A study by Kang et al. [144] reported that Swamp buffalo fed with a high concentrate (160 g/kg DM) supplement ratio had constant ruminal pH (average 6.59 to 6.61) and temperature (average 39.1 to 39.3 °C), but had increased values of ammonia, total VFA, propionic acid, butyric acid of 9.8 vs. 13.8 mg/dL, 20.1 vs. 26.2 mol/100 mol and 10.7 vs. 12.3 mol/100 respectively, when compared to animals fed with low concentrate (120 g/kg DM).

Carbohydrate in the diet is mainly characterized by the proportion of non-structural (NSC) and structural carbohydrate [145,146]. In the rumen, the NSC are initially utilized by the rumen microbes and are degraded quickly to produce volatile fatty acids [147]. Sutton et al. [148] confirmed that high NSC in the concentrate diet resulted in higher propionate concentration, while McCarthy et al. [149] also concluded that increasing the content of ruminal fermentable starch enhanced the total volatile fatty acid (TVFA) concentration. In fact, decreased ruminal pH was also reported following high intake levels of dietary crude protein, and this was attributed to the increased ruminal total VFA output [150,151]. Ruminal ammonia-nitrogen (NH_3_-N) is primarily derived from ruminal degradable proteins and is used for the synthesis of microbial protein [152,153]. An increase in ruminal NH_3_-N content occurs following an increase in dietary CP levels [152] and is largely attributed to the increased ruminal CP degradability [154]. However, an increase in dietary concentrate or carbohydrate is not a successful strategy to mitigate either the enteric methane production or ammonia emission from the manure. Therefore, incorporating supplemented concentrate with bypass fat has the potential of reducing the methane and ammonia productions.

Unfortunately, a study by Budi et al. [155] found that the increased level of bypass fat significantly reduced the in vitro dry matter degradability, but the levels of TVFA and ammonia nitrogen remained constant, while Naik et al. [156] revealed that following in vitro fermentation, the levels of TVFA and ammonia nitrogen were much higher in animals fed with a diet with concentrate and bypass fat compared to those fed without the supplement. Saijpaul et al. [157] recommended that the high level of bypass fat supplementation could substitute up to 40% of natural fat in a concentrate mixture (6% natural fat) contained in total mixed rations (50:50 roughage to concentrate) with no changes in in vitro fermentation parameters of TVFA, total nitrogen and ammonia. Other studies also reported that supplementation of bypass fat between 5% and 15% in buffalo diet had no adverse effect on the in vivo rumen fermentation characteristics such as pH, NH_3_-N and VFA levels [158,159,160]. The specific ingredient of dietary buffer in calcium salt bypass fat functioned to maintain ruminal pH and to minimize the rate of dissociation of calcium salts in the rumen [82], thus the rate of biohydrogenation was potentially reduced. In summary, supplementation of solely concentrate in a basal diet could affect the buffalo rumen fermentation characteristics but no adverse effects were found on the reported parameters when buffaloes fed given the mixture of concentrate and bypass fat in the diet.

### 5.4. Rumen Microbial Populations

It is also known that the feed and feeding regime in ruminants may also significantly affect the ruminal microbial community. Indeed, it has been demonstrated that the composition of the rumen bacterial population significantly correlated with feed efficiency [161,162]. A study showed that changes in the structure of the ruminal microbial population potentially promoted feed efficiency, intake and the average daily gain of ruminants [163]. The microorganisms also often provided the host ruminant with nutrients to produce energy [164]. Thus, increased ruminant production could be achieved by using proper feeding formulation and management that were able to manipulate the ruminal microbes and ecosystem. For example, a proper ratio of supplemented feed in the diet that provided adequate energy and protein, allowed for an optimal ruminal fermentation process that maximized production efficiency while decreasing energy loss such as methane that polluted the environment [165]. Indeed, there were also reports on the effects of dietary changes involving protein and energy on rumen microbial population and the rumen environment in ruminants [166].

The effects of dietary energy and protein supplementation on the rumen microbial population have been studied extensively in sheep, goat and cow, but less so in buffaloes. Indeed, a study by Dahllöf et al. [167] has reported that dietary modifications in cattle feeding have major effects on the communities of rumen bacteria. Faniyi et al. [168] also stated that a shift in the ruminant diet from forage-based to a high-concentrate diet resulted in significant alterations of the ruminal environment and rumen bacterial population structure. Indeed, various studies also showed that there were clear changes in the structure of the rumen bacterial population as the dietary forage to concentrate ratio gradually increased from 80:20 to 60:40 or even to 20:80, with increases in Proteobacteria and decreases in Firmicutes [168,169,170,171,172]. The increased abundance of Proteobacteria during high-concentrate diets was suggestive of an increased need for bacterial species that could metabolize the newly available fermentable carbohydrates [169], thus favoring the growth of amylolytic and other starch-digesting bacterial species and reducing the number of cellulolytic bacteria. This suggested that when animals were shifted from a forage diet to a high-concentrate diet, the microbial diversity in terms of the different species numbers remained but the composition or the species makeup changed drastically in order to adapt to the new rumen environment.

*Fibrobacter succinogenes,* a fibrolytic bacterium that digests fiber, was reported to be gradually decreased as animals were adapted to a high-concentrate diet, and their numbers were 40-fold lower than in those animals fed on hay [169]. A study by Tajima et al. [173] reported a 20-fold decrease in the *Fibrobacter* population size by day 3 and a 57-fold decrease by day 28 in animals on high-concentrate diets. The population of *Butyrivibrio fibrisolvens*, another fibrolytic bacterium with high affinity toward maltose and sucrose, also declined 20-fold during adaptation to a high-concentrate diet [172]. Due to the ability of this species to use both cellulose and starch, the *Butyrivibrio fibrisolvens* population showed a slight decrease in the rumen environment [169]. However, the drop in the population of *Butyrivibrio fibrisolvens* during a diet with a high proportion of concentrate (30:80) might be because of pH changes due to the increased number of fermentable substrates present within the rumen. This was consistent with the findings of a recent study which showed that the population of *Butyrivibrio fibrisolvens* increased in high-fiber diets and decreased in high-energy diets [174,175].

Changes in bacterial populations with increasing dietary crude protein were attributed to the increased ruminal amino acids, peptides, branched chain VFA and NH_3_-N [176,177]. Moreover, it was reported by Wang et al. [178] that increased dietary crude protein would enhance ruminal microbial growth, particularly of the bacterial populations in the rumen fluid that consisted of *Ruminococcus albus, Ruminococcus flavefaciens, Butyrivibrio fibrisolvens, Prevotella ruminicola, Fibrobacter succinogenes* and *Ruminobacter amylophilus*. In general, the improved microbial enzyme activity has a significant relationship with the increase of the bacterial populations in the rumen [143]. In particular the activities of cellobiase, xylanase, pectinase, carboxymethyl-cellulase, pectinase, protease and α-amylase increased following an increase in dietary protein. However, another researcher showed that lambs fed with a diet containing crude protein levels from 111.7 to 143.6 g/kg DM did not show any effect on the rumen microbial population of *R. albus, R. flavefaciens* and *F. succinogenes* [179]. These contradictory findings might be due to the differences in the animals studied and the dietary composition [151,179].

### 5.5. Qualities of Carcass and Buffalo Meat and Their Implications on Human Health

Nutrition is a dominant component of livestock production systems that influences several aspects of meat quality. Consumer demands on meat quality have motivated the meat producers to focus on the nutritional aspects of livestock rearing since carcass and meat qualities are affected by the amount and type of nutrient intake. These include dressing yield, meat to bone ratio, protein to fat ratio, fatty acid composition, caloric value, color, physicochemical and processing properties, shelf life and sensory attributes [180].

Dressing percentage is one of the important parameters that reflects the potential meat yield from an animal. Usually, the weight of hot carcass is used to compute the dressing percentage. When the weight of cold carcass is used, the dressing percentage is less due to the chilling shrinkage that ranges between 3% and 4.5% of the initial weight of the hot carcass for buffaloes at the age of 6 months up to 4 years [181]. Other than chilling, a feeding diet with supplementation might inconsistently influence the carcass quality. Anjaneyulu et al. [182] reported that supplemented dietary protein did not affect the carcass composition of male buffalo. However, the dressing percentages and yields of the lean meat were higher when buffaloes were fed a high-energy diet compared to those fed a low-concentrate diet [112]. Nevertheless, based on the current information, it was concluded that feed supplementation had little effect on the carcass quality of buffaloes.

Meat quality is a major factor that is used for marketing the product [183]. The meat quality is evaluated through physical, biochemical, histological and sensory analyses. The nutritional composition and pH of meat are parts of the biochemical analyses used in assessing the meat quality, which contribute to the edibility or the desirability of the product [184]. Three crucial factors can affect the quality and composition of the meat produced [185]. They are: (1) the feedstuffs and proportion of the diet used for feeding the animal, (2) types of diet, supplement, breed or genetic cross of the animal and (3) the age at which the animal is slaughtered. Meanwhile, diet has been shown to be one of the most important environmental factors that influences the carcass yield, meat cutability and quality [186,187]. The effects of feeding on meat quality are generally studied in terms of the content and composition of the lean and fat tissues, and the subsequent effects on the nutrient content of protein, fat, energy and moisture content.

In particular, the total fatty acid content and the types of fatty acid found in meat have major influences on the meat quality and acceptability of the meat by consumers [187]. Feeding a high-energy diet potentially affects the rate of conditioning and consequently, the carcass composition, conformation, meat yield and meat and fat quality [188].

Carabeef, also known as buffalo meat, is considered to be a highly nutritious and valuable food. It is a source of high biological value of protein, omega 3 and omega 6 fatty acids and low in fat and cholesterol levels [189]. However, different nutrient contents of buffalo meat have been reported due to different feeding regimes [190]. Lambertz et al. [191] proposed a concentrate supplementation at the rate of approximately 1.5% of body weight to enhance the carcass characteristics of Swamp buffaloes via expressing superior dressing percentage, better muscling and redder meat, with higher contents of protein and fat. Furthermore, addition of a fat supplement enables facilitated absorption of liposoluble nutrients, making it possible to modify the meat fat composition according to consumers’ demand [192]. Moreover, the high energy levels allow the increase of pulp proportion in the diet of fattening animals, which act as precursors of the fatty acid responsible for the lack of consistency of fat from carcasses [193].

## 6. Conclusions

From the available literature, it can be summarized that supplementation of concentrate and bypass fat in a potential buffalo diet is very important to overcome the problem of negative energy balance without adversely affecting the dry matter intake, rumen fermentation, blood metabolites and rumen microbial populations. Supplementation of concentrate and bypass fat provides additional benefits due to improved ruminant body weight, body condition score and the economics of the ruminant industry. Further research is necessary to find out the effects of supplementation with concentrate and bypass fat on growing buffaloes fed with various types of basal rations at different productive levels.

## Data Availability

Not applicable.

## Abbreviations

CP	Crude protein
RUP	Rumen undegradable protein
kg	Kilogram
g	Gram
mg	milligram
ADG	Average daily gain
TVFA	Total volatile fatty acid
DM	Dry matter
BW	Body weight
BCS	Body condition score
NEFA	Non-esterified fatty acid
dl	Deciliter
GH	Growth hormone
IGF-1	Insulin-like growth factor- 1
GHR	Growth hormone receptor
BUN	Blood urea nitrogen
